# Retrograde Balloon-Assisted Deep Enteroscopy in the Diagnosis of Metastatic Melanoma

**DOI:** 10.1155/2021/5572230

**Published:** 2021-06-30

**Authors:** William Barge, Mark R. Albertini, Christopher Cold, Daniel Abbott, Deepak Gopal

**Affiliations:** ^1^University of Wisconsin School of Medicine and Public Health (UWSMPH), Madison, Wisconsin, USA; ^2^Division of Hematology, Medical Oncology & Palliative Care, UW Carbone Cancer Center Comprehensive Melanoma Clinic, UWSMPH, Madison, Wisconsin, USA; ^3^Department of Pathology and Laboratory Medicine, UWSMPH, Madison, Wisconsin, USA; ^4^Division of Surgical Oncology, Department of Surgery, UWSMPH, Madison, Wisconsin, USA

## Abstract

A 74-year-old male with a history of metastatic melanoma presents with persistently abnormal small bowel findings on PET-CT scan. The patient had persistent FDG uptake near the ileocolic junction on imaging, concerning for metastatic melanoma. Capsule endoscopy demonstrated ulcerated mucosa in the distal ileum. This area was biopsied and tattooed via retrograde double-balloon enteroscopy to confirm the diagnosis of metastatic melanoma and facilitate subsequent small bowel resection. The case illustrates a unique case of metastatic melanoma to the small bowel and the utility of capsule endoscopy and balloon-assisted enteroscopy to assist in diagnosis and management of metastatic disease.

## 1. Introduction

A 74-year-old male with a medical history of metastatic melanoma presents to the outpatient gastroenterology clinic due to persistently abnormal small bowel findings on positron emission tomography-CT (PET-CT) scan. He is otherwise asymptomatic and has no complaints. The following case illustrates a unique and unusual case of metastatic melanoma to the small bowel and the utility of both capsule endoscopy and balloon-assisted deep enteroscopy to assist in diagnosis and preoperative management.

## 2. Case Description

A 74-year-old male with a history of previously excised melanoma metastatic to a mesenteric lymph node who had been in remission for 2 years before presenting for additional evaluation of persistently abnormal small bowel findings on a surveillance PET-CT scan. Patient reported feeling well without complaints. He had a medical history of hypothyroidism, GERD, lymphocytic colitis on loperamide therapy, and metastatic melanoma. He was originally diagnosed with melanoma on his back ten years prior to this presentation. His melanoma history includes a left axillary recurrence over 3 years prior to this presentation, initial adjuvant therapy with pembrolizumab that was discontinued after 2 doses due to lymphocytic colitis, and laparoscopic resection of a metastatic mesenteric lymph node that was excised 2 years prior to this presentation. The patient's other surgical, family, and social history are unremarkable.

**Figure 1:**
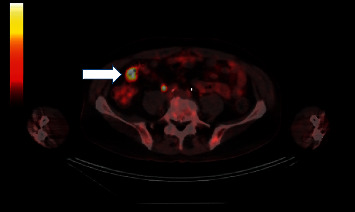
PET (positron emission tomography) scan with persistent and increasing FDG uptake in distal small bowel (arrow).

**Figure 2:**
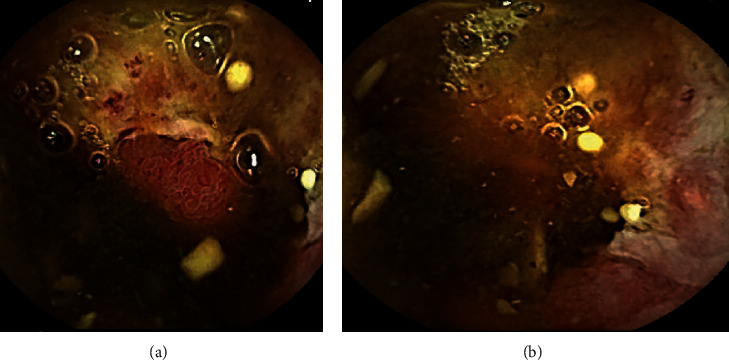
(a) Capsule endoscopy image of erythematous, ulcerated, and congested area of mucosa presumed to be localized to the distal ileum at 6 hours, 26 minutes, and 12 seconds. (b) Additional image of the aforementioned area located in distal ileum on capsule endoscopy.

**Figure 3:**
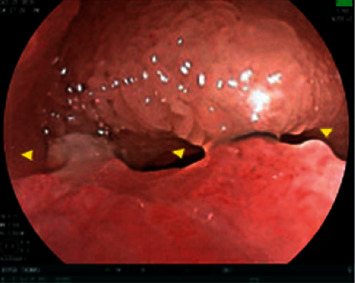
Ulcerated and inflamed nodular area (15 mm × 20 mm) in distal ileum identified on DBE and measured at 95 cm from the ileal valve. This area was subsequently biopsied and tattooed with carbon black in preparation for resection.

**Figure 4:**
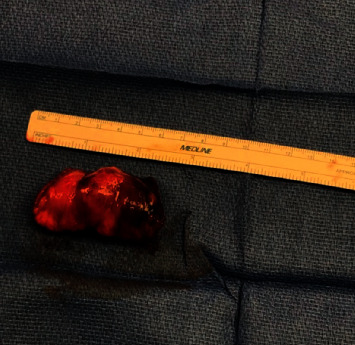
Surgical specimen from laparoscopic-assisted small bowel resection of the tattooed site (Figure 3).

**Figure 5:**
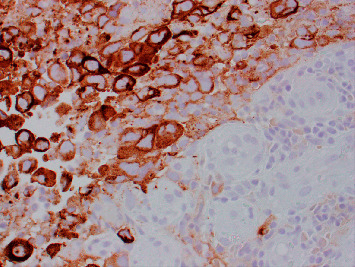
H&E staining of surgical specimen (Figure 4) with positive stain for HMB45 and Melan-A, suggesting diagnosis of melanoma.

Since his previous lymph node excision two years prior to this presentation, multiple surveillance PET scans had shown intense focal FDG uptake in the distal small bowel, deemed to likely be inflammatory in nature although melanoma recurrence and metastasis remained a possibility. The patient underwent PET-CT scan in the weeks prior to presentation which showed interval increased FDG uptake at the distal small bowel near the ileocolic junction (Figure 1). This finding featured an SUVMax of 16.4, previously noted to be an SUVMax of 7.4 at prior PET/CT. Given recurrent findings, patient underwent capsule endoscopy which revealed ulcerated, nodular, congested, and erythematous mucosa at 6 hours, 26 minutes, and 12 seconds past swallowing capsule, with location deemed likely in distal ileum (Figures 2(a) and 2(b)). Given this finding, patient underwent retrograde double-balloon enteroscopy (DBE) which showed ulcerated, edematous mucosa in the distal ileum at 95 cm from ileocecal valve (Figure 3), with biopsies consistent with metastatic melanoma as confirmed by the presence of melanin in tumor biopsy and positive staining. The site of the lesion was marked with SPOT tattoo to allow for intraoperative localization. Patient underwent surgical resection of this area (Figure 4), which also revealed the second area of ileal metastasis (2.8 cm of the ileum) separate from the tattooed area (2.6 cm), and seven lymph nodes removed were negative for metastatic melanoma. The surgical resection specimens also demonstrated the presence of melanin and had positive staining for melanoma (Figure 5). Patient underwent surveillance DBE 8 months after initial procedure, which showed patent anastomosis and normal mucosa up to 110 cm from the ileocecal valve. Patient has undergone PET/CT scans every 3 months following surgical resection, and he was without evidence for recurrent or metastatic disease at his most recent clinic evaluation one year from surgery.

## 3. Discussion

This case illustrates the utility of capsule endoscopy to identify small bowel metastasis, an atypical use in the current practice of capsule endoscopy. Furthermore, this case details how retrograde double-balloon enteroscopy, in combination with diagnostic modalities such as PET scans and capsule endoscopy, can assist in the diagnosis of distal small bowel disease including metastasis. This case also illustrates how SPOT tattoo utilization may assist with intraoperative surgical localization of suspicious lesions. A prior small study (total of 8 patients) has also shown that capsule endoscopy may reveal metastatic small bowel disease that general endoscopy, push enteroscopy, small bowel follow-through imaging, CT scan, or PET may not detect [1]. Other studies have showed that capsule endoscopy may also be better at identifying extent of small bowel metastasis than other imaging modalities [1, 2]. While metastatic melanoma in the small intestine is a known and common phenomenon [3–5], small bowel metastasis has been detected clinically in only 2–5% of patients with malignant melanoma [6]. Although these lesions are typically resected surgically for diagnosis, our case illustrates the role of initial small bowel endoscopic imaging to assist in localization to facilitate intraoperative therapy such as the combination of capsule endoscopy and retrograde double-balloon enteroscopy and is a practical and feasible method to evaluate for metastatic disease in the distal small bowel. Accurate preoperative localization of small bowel metastasis is even more important considering metastatic melanoma to the small bowel can be complicated by obstruction or perforation, and surgical resection is associated with prolonged survival and symptom improvement and remains an important treatment of metastatic melanoma to the small bowel [7, 8].

A literature review illustrates that little research has been completed regarding retrograde double-balloon enteroscopy in the diagnosis of metastatic melanoma. One study that involved both anterograde and retrograde DBE revealed that preoperative DBE can modify the surgical approach to malignant tumors of the small bowel [9], although few of the included cases involved retrograde endoscopy. The other literature involving DBE in the diagnosis of metastatic melanoma is at the case report level [10], and none involve retrograde endoscopy. Our study is a unique illustration of how retrograde DBE in combination with capsule endoscopy can be used for the preoperative localization of metastatic melanoma of the small bowel.

## Data Availability

No compiled data were used to support this study.
